# Supplementary material for: Florigen activation complex forms via multifaceted assembly in Arabidopsis
Source: Nature. 2025 Nov 12;648(8094):686–95. doi: 10.1038/s41586-025-09704-6 (PMC12711580; doi:10.1038/s41586-025-09704-6)
Supplement: Supplementary file 1 — Supplementary Text and Figs. 1–4 containing additional results and discussion on the formation of the FAC, including EMSA, nuclear localization and DNA binding of FD, phosphorylation of FD at Thr282 and its interaction with 14-3-3 proteins, structural flexibility of the FD C-terminal region, and evolutionary analysis of 14-3-3, bZIP and PEBP families. References cited in the Supplementary Text. [file 41586_2025_9704_MOESM1_ESM.docx]

Supplementary information for

**Florigen activation complex forms via multifaceted assembly in Arabidopsis**

Gao and Ding et al.

Corresponding author:

He Gao, [gao@mpipz.mpg.de](mailto:gao@mpipz.mpg.de); George Coupland, [coupland@mpipz.mpg.de](mailto:coupland@mpipz.mpg.de)

**The PDF file includes:**

Supplementary Text

References

Supplementary Figure 1−4

**Other Supplementary Material for this manuscript includes the following:**

Supplementary Table 1−4 are provided as separate Excel files.

**Supplementary Text**

**The FAC formation examined by EMSA experiments *in vitro***

Consistent with the gel-filtration results, EMSA experiments showed a more pronounced supershift when FT was combined with MBP-FDc^T282E^ (where the FD C terminus is solubilized by MBP) and GRF7 incubated with DNA, compared to the supershift with only GRF7 and MBP-FDc^T282E^ proteins (Extended Data Fig. 4j, left panel). No supershift was detected when FT^Mu1^ was combined with MBP-FDc^T282E^ and GRF7. Nevertheless, a supershift was still detected when MBP-FDc^T282E^ was combined with GRF7^Mu1^ and FT, GRF7 and FT^Mu2^ or GRF7^Mu1^ and FT^Mu2^. Notably, a supershift with stronger DNA binding was still detected when MBP-FDc^T282E^ and FT proteins were combined, compared with MBP-FD^T282E^ alone with DNA (Extended Data Fig. 4j, right panel). This effect was not limited to the *SEP3* locus, because the same result was achieved with a second FD target, *LFY* (Extended Data Fig. 4k). Collectively, our *in vitro* data suggest that under stringent gel-filtration conditions, both FT interfaces, which interact with the DNA in the DNA-bound FD−14-3-3 complex (interface 1) or 14-3-3 (interface 2), respectively, are necessary for its recruitment to the FAC, whereas under less stringent EMSA conditions, the formation of interface 1 that interacts with DNA in the DNA-bound FD−14-3-3 complex plays a more substantial role and alone is sufficient to recruit FT.

**The FD basic domain facilitates nuclear localization and DNA binding**

The basic domain of bZIPs is essential for DNA binding and FD binds to the G-box sequences of its target genes such as *SEP3*. Intriguingly, one nuclear localization signal (NLS) was predicted within the basic domain at the N-terminus of the leucine zipper (ZIP) of FD, and this motif is highly conserved among Group A bZIPs in Arabidopsis (Extended Data Fig. 5a and Extended Data Fig. 12 c). Mutations of nine basic amino acids within the predicted NLS in mVenus-FD (Mu1) greatly attenuated its nuclear localization *in vivo* and impaired its function in promoting flowering (Extended Data Fig. 5b−f; Supplementary Table 1). To understand how FD binds to DNA, a structural model of the FD^bZIP^−DNA^SEP3^ complex was made (Methods). This model showed that several of the amino acids (R217, R225, R232, R234) of FD were predicted to interact with DNA from the *SEP3* promoter that includes FD binding site *in vivo* (Fig. 3n and Extended Data Fig. 5k,l). MBP-FD^161-285 (T282E)^ and MBP-FD Mu1^161-285 (T282E)^ proteins were purified and their specific binding to DNA was analyzed by electrophoretic mobility shift assays (EMSA) using *SEP3* DNA probes (Extended Data Fig. 5m). The DNA fragment containing one G-box was bound by FD^161-285 (T282E)^, but the binding of FD^Mu1^ was strongly impaired. This impairment is similar to the effects observed with a G-box mutation in the DNA. Therefore, the basic domain of FD has a dual function in conferring nuclear localization and DNA binding, which is a common feature of other bZIP proteins^1,2^.

**Phosphorylation of FD at Thr 282 enables *in vivo* interaction with 14-3-3 proteins for the promotion of flowering at the shoot apex**

First, the temporal expression patterns of FD and 14-3-3 proteins were compared by RNA-sequencing^3^ (Extended Data Fig. 2a) and confocal microscopy using genomic fusions of *gFD::mVenus-FD*, *gGRF2::mScarlet1-GRF2* (14-3-3ω), *gGRF6::mScarlet1-GRF6* (14-3-3ƛ), *gGRF7::mScarlet1-GRF7* and *gGRF8::mScarlet1-GRF8* (Extended Data Fig. 2b−e). Levels of *FD* mRNA and FD target genes showed a gradual increase, peaking after the floral transition (16LDs), whereas *GRF* mRNA levels remained constant at different developmental stages. Moreover, GRF2, 6, 7, and 8 fluorescent protein fusions were widely detected, and co-localized with FD in both apical and leaf tissues throughout various developmental stages, albeit with slight spatiotemporal variations. Moreover, GRF2, GRF6, and GRF7 accumulated in the cytosol and nucleus, whereas GRF8 was notably enriched in the nucleus. Therefore, FD and 14-3-3 proteins are broadly co-expressed in the SAM before, during and after floral transition under LDs.

Phosphorylation of residue(s) in the conserved carboxy-terminal motif (C4, SAP) of FD and other Group A bZIP proteins has been proposed as a potential recognition site for 14-3-3s (Extended Data Fig. 5a)^4,5^, but this has not been demonstrated *in vivo*. To identify phosphorylated residues of FD *in vivo*, 3HA-mCherry-FD protein was immunoprecipitated from *gFD::3HA-mCherry-FD; fd-3*^6^ seedlings undergoing floral transition and analyzed by MS (10-13 LDs; Fig. 1a). Phosphorylated T282 was detected (Extended Data Fig. 6a), and the phosphorylation appeared to occur independently of developmental stage or photoperiodic conditions (Supplementary Table 2). However, no unphosphorylated T282 peptides were found, suggesting that phosphorylated T282 is the dominant form *in vivo*. Furthermore, we examined the impact of phosphorylation of FD on floral induction by generating *fd-3* transgenic plants expressing genomic (containing all regulatory sequences; Method) fusions of an N-terminal epitope 3HA-3Flag tagged FD^WT^ and mutants encoding mimic phosphorylated FD^T282E^ and non-phosphorylatable FD^T282A^. A *gFD::3HA-3Flag-FD^WT^* construct fully rescued the late flowering of *fd-3* mutants in the T1 generation. By contrast, a similar construct expressing a form of FD that cannot be phosphorylated (*gFD::3HA-3Flag-FD^T282A^*) failed to rescue *fd-3* (Extended Data Fig. 6b and 7a; Supplementary Table 1). Therefore, these results suggest that the dominant phosphorylated FD form at T282 is the primary active form responsible for floral induction. In support of this idea, plants expressing phosphorylation-mimic FD (*gFD::3HA-3Flag-FD^T282E^*) did not flower earlier than Col-0 and 3HA-3Flag-FD^WT^, in contrast to findings from a previous study^5^.

14-3-3 proteins recognize phosphomotifs^7^. To identify potential FD partners *in vivo*, immunoprecipitation-Mass Spectrometry (IP-MS) was performed on nuclear protein extracts from seedlings undergoing floral transition using a functional genomic fusion of transgenic *gFD::3HA-mCherry-FD; fd-3* line. A total of 75 high confidence (FDR=0.05) protein–protein interactors (PPIs) of FD were identified in biological triplicate (Supplementary Table 3), including three Group A bZIP TFs, ABA-RESPONSIVE ELEMENT BINDING PROTEIN (AREB) 1, 2 and 3. Among these, AREB3 was previously found to interact with FD and to be partially genetically redundant with it in inducing flowering^6^. Notably, the PPIs included ten (13.3%) 14-3-3 paralogs, also called GENERAL REGULATORY FACTOR (GRF). Therefore, FD and 14-3-3s, two key components of the proposed FAC, interact *in vivo* in Arabidopsis seedlings.

The Group A bZIP ABA RESPONSIVE ELEMENT-BINDING FACTOR 3 (ABF3) protein abundance was proposed to be enhanced by 14-3-3 binding in Arabidopsis^8^. However, mRNA and protein expression levels of 3HA-3Flag-FD^T282A^ were comparable to those of 3HA-3Flag-FD^WT^ (Extended Data Fig. 7a−c). Accordingly, protein structure modeling predicted a weak or no interaction between the proteins without FD T282 phosphorylation (Extended Data Fig. 6c). Moreover, IP-MS experiments revealed that 14-3-3 proteins were not co-immunoprecipitated by 3HA-3Flag-FD^T282A^, in contrast to 3HA-3Flag-FD^WT^ (Extended Data Fig. 6d−f; Supplementary Table 2). We next examined the interaction between 14-3-3 and FD proteins *in vitro* using *Escherichia coli*-purified GRF7 and FD proteins. The maltose binding protein (MBP) was fused to the FD N-terminus to enhance its solubility. Efficient phosphorylation of FD did not occur in *E. coli* cells (Supplementary Table 2); therefore, MBP-fused phosphorylation mimic (FD^T282E^) and non-phosphorylatable (FD^T282A^) FD mutant proteins were purified. Size exclusion chromatography (gel-filtration) showed that GRF7 strongly interacted with FD^T282E^ *in vitro*, but not with FD^T282A^ (Extended Data Fig. 6g−j). Taken together, these data provide *in vivo* evidence of FD phosphorylation at T282 and show this residue is required for the interaction with 14-3-3 proteins *in vivo* and *in vitro*, and strongly suggest that this interaction is required for FD^WT^ activity during floral induction.

**Binding of 14-3-3 proteins to FD enhances DNA binding by regulating the flexibility of the C13 region**

The presence of GRF7 not only facilitated the dimerization of MBP-FDc^T282E^ but also enhanced its DNA binding (Fig. 4, Extended Data Fig. 7 and Extended Data Fig. 9). Our protein structure modeling suggests that the binding of GRF7 to FD may limit the flexibility of its C-terminus by inducing conformational changes in a 13-amino acid region (C13) between the bZIP and 14-3-3 binding site, a region not conserved in the Group A bZIPs (Extended Data Fig. 5a and Extended Data Fig. 10a,b). Structural models were created to explore the impact of C13 on DNA binding, involving C13 mutants with all residues in C13 of FDc^WT^ substituted with alanines (A) and incombination with an additional substitution of T282E (Mu7) into T282A (Mu8). In the models, C13 of FDc^WT^ (amino acids 215−285) constituted a flexible segment between the bZIP domain and the 14-3-3-binding region, whereas Mu7 and Mu8 formed an extended α-helix from the bZIP domain through the C13, potentially reducing flexibility at the FD C-terminus. EMSA experiments were then performed with purified MBP-FDc^T282E^, MBP-FDc^M7^, and MBP-FDc^Mu8^ proteins. Both C13 mutants significantly increased DNA-binding affinity compared with FDc^T282E^ when GRF7 was absent (Extended Data Fig. 10c). Therefore, these results suggest that the binding of 14-3-3 proteins to FD not only dimerize the FD C-terminus but also limit the flexibility of the adjacent C13 region, thereby enhancing DNA binding. To examine the effect of C13 on FD activity *in* *planta*, *gFD::3HA-3Flag-FD^Mu7(T282)^; fd-3* and *gFD::3HA-3Flag-FD^Mu8(T282A)^; fd-3* transgenic plants were constructed. Albeit slightly weaker than wild type (WT) FD, FD^Mu7^ significantly rescued the late-flowering phenotype of *fd-3* mutants (Extended Data Fig. 10d and Supplementary Table 1), indicating that the C13 mutation largely preserves FD activity *in vivo*.

However, these data also suggest that the flexibility of the WT protein at the C-terminus enhances its activity *in vivo*. Similar to mVenus-FD^WT^ (Fig. 4), mVenus-FD^Mu7^ appeared evenly distributed in the nuclei (Extended Data Fig. 10e, left panel), suggesting that Mu7 could still be bound by 14-3-3s *in vivo*. By contrast, mVenus-FD^Mu8^ that includes the T282A mutation formed large nuclear condensates (Extended Data Fig. 10e, right panel) and did not promote flowering (Extended Data Fig. 10d and Extended Data Fig. 11), although it bound DNA more strongly *in vitro* when solubilized by the MBP tag. Consistent with this notion, our gel-filtration revealed robust GRF7 binding to Mu7^T282E^ but not Mu8 *in vitro* (Extended Data Fig. 11). Collectively, these data demonstrate that 14-3-3 proteins have multifaceted roles in enhancing DNA-binding by FD, via promoting dimerization and regulating the flexibility of the C13 region. However, it appears that the prevention of large condensates formation by FD, facilitated by 14-3-3 proteins, has a significant impact in regulating FD activity to promote flowering.

**Evolutionary origins and conservation of 14-3-3, bZIP, PEBP families, and emergence of the florigen activation complex in plant evolution**

To extend our understanding of the co-evolution of 14-3-3, bZIP and PEBP proteins in the plant kingdom, we constructed phylogenetic trees of the three gene families potentially present in red algae and green plant genomes (Extended Data Fig. 12; Supplementary Table 3). 14-3-3 and bZIP genes were found at the base of the phylogenetic trees, confirming the algal origin of the two families. Three major bZIP groups were identified from charophytes (green algae), and the potential phosphomotifs at the C-terminus to which 14-3-3s bind first appeared in charophytes in the lineage leading to Group A bZIPs that contain FD. The C-terminal motif to which 14-3-3 binds is therefore an ancient feature of one lineage of bZIPs in the plant kingdom and this appears to have arisen in green algae. In addition to the bZIP and C-terminal 14-3-3 recognition domain, some conserved motifs at the N-termini of bZIP proteins were identified, particularly the RXXS/T motif that was reported in higher plants to be phosphorylated in response to ABA^9^ and was already present in the charophytes. Although widely present in Group A bZIPs, the N-terminal RXXS/T motifs were lost in the FD/FDP subclade, which became fixed in gymnosperms and angiosperms. The functional residues of 14-3-3 proteins are highly conserved in charophytes and throughout the plant kingdom (Supplementary Fig. 5c and Supplementary Table 3). PEBP genes were found at the base of green plants, encompassing two branches – *MFT1* and *MFT2*. MFT1 is present only in early green plants (from Charophytes to ferns), whereas MFT2 is found throughout the entire green plant lineage. Notably, the *FT* and *TFL1*/*CEN/BFT*(*TCB*) clades emerged for the first time in gymnosperms and are highly conserved in the entire seed plant lineage (gymnosperms and angiosperms). The residues in the FT tail that contribute to its recruitment to the DNA−FD−14-3-3 complex are highly conserved among angiosperms, emphasizing their crucial role in mediating the assembly of the FAC in flowering plants. Overall, these analyses suggest that the regulation of bZIP dimerization and specificity that we described for FD and 14-3-3 proteins likely evolved early in the green plant lineage around the time of the appearance of charophytes and the emergence of *FT* genes in angiosperms is likely to be determinant of the formation of the FAC, and thereby of the photoperiodic control of floral induction.

**References**

1 Varagona, M. J. & Raikhel, N. V. The basic domain in the bZIP regulatory protein Opaque2 serves two independent functions: DNA binding and nuclear localization. *Plant J* **5**, 207-214, doi:10.1046/j.1365-313x.1994.05020207.x (1994).

2 Schutze, K., Harter, K. & Chaban, C. Post-translational regulation of plant bZIP factors. *Trends Plant Sci* **13**, 247-255, doi:10.1016/j.tplants.2008.03.002 (2008).

3 Cerise, M. *et al.* Two modes of gene regulation by TFL1 mediate its dual function in flowering time and shoot determinacy of Arabidopsis. *Development*, doi:10.1242/dev.202089 (2023).

4 Taoka, K. *et al.* 14-3-3 proteins act as intracellular receptors for rice Hd3a florigen. *Nature* **476**, 332-335, doi:10.1038/nature10272 (2011).

5 Collani, S., Neumann, M., Yant, L. & Schmid, M. FT Modulates Genome-Wide DNA-Binding of the bZIP Transcription Factor FD. *Plant Physiol* **180**, 367-380, doi:10.1104/pp.18.01505 (2019).

6 Martignago, D. *et al.* The bZIP transcription factor AREB3 mediates FT signalling and floral transition at the Arabidopsis shoot apical meristem. *PLoS Genet* **19**, e1010766, doi:10.1371/journal.pgen.1010766 (2023).

7 Pennington, K. L., Chan, T. Y., Torres, M. P. & Andersen, J. L. The dynamic and stress-adaptive signaling hub of 14-3-3: emerging mechanisms of regulation and context-dependent protein-protein interactions. *Oncogene* **37**, 5587-5604, doi:10.1038/s41388-018-0348-3 (2018).

8 Sirichandra, C. *et al.* The Arabidopsis ABA-activated kinase OST1 phosphorylates the bZIP transcription factor ABF3 and creates a 14-3-3 binding site involved in its turnover. *PLoS One* **5**, e13935, doi:10.1371/journal.pone.0013935 (2010).

9 Jakoby, M. *et al.* bZIP transcription factors in Arabidopsis. *Trends Plant Sci* **7**, 106-111, doi:10.1016/s1360-1385(01)02223-3 (2002).

**Supplementary figures**


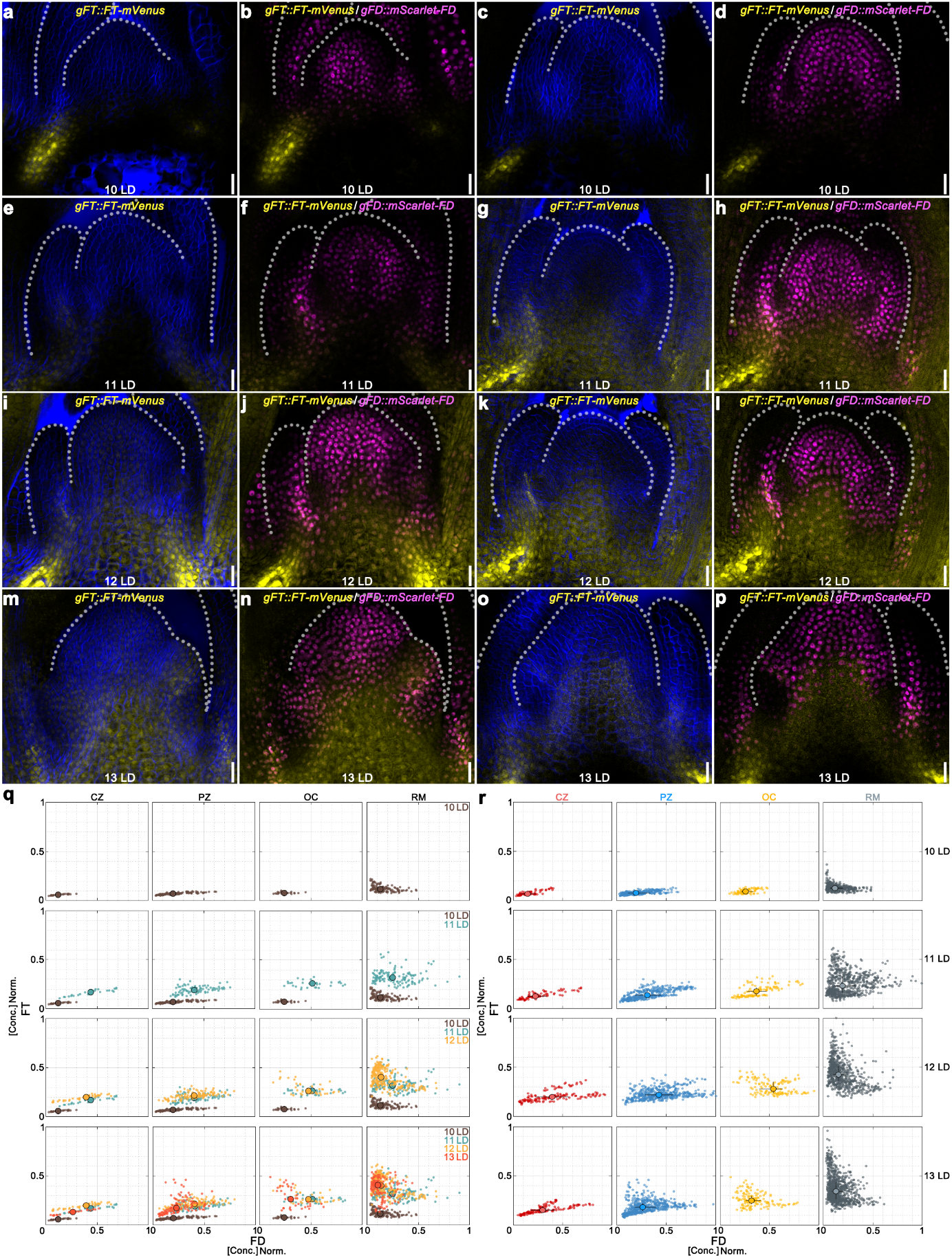


**Supplementary Figure 1**

**Distribution of FT and its co-localization with FD at the SAM during floral transition.** **a**−**p**, Confocal images of additional independent shoot apical meristems co-expressing *gFT::FT-mVenus* and *gFD::mScarlet1-FD*. Each row shows two independent meristems analyzed for each time point (10 to 13 LDs). Scale bars = 20 μm. **q**. Normalized FT *vs* FD nuclear concentration for single meristems shown in Fig. 1 **f**−**i** at the Central Zone (CZ), Peripheral Zone (PZ), Organising Center (OC) and Rib Meristem (RM). First row: SAM at 10 LDs; second row, SAM at 10 and 11 LD; third row, SAM at 10, 11 and at 12 LD. Fourth row: same as third row, including 13 LD-SAM. Big circles represent the median (FD, FT) nuclear concentration for all nuclei of a single SAM within each meristematic regions. **r**. Normalized FT *vs* FD nuclear concentration for all meristems analyzed in Fig. 1 j and k at the Central Zone (CZ), Peripheral Zone (PZ), Organising Center (OC) and Rib Meristem (RM). First, second, third and fourth rows correspond to SAM data for 10, 11, 12 and 13 LDs, respectively. Big circles represent the median (FD, FT) nuclear concentration from the individual SAM median nuclear (FD, FT) concentrations within each meristematic region. Error bars are the FT and FD interquartile range (IQR) of the previous median distribution.


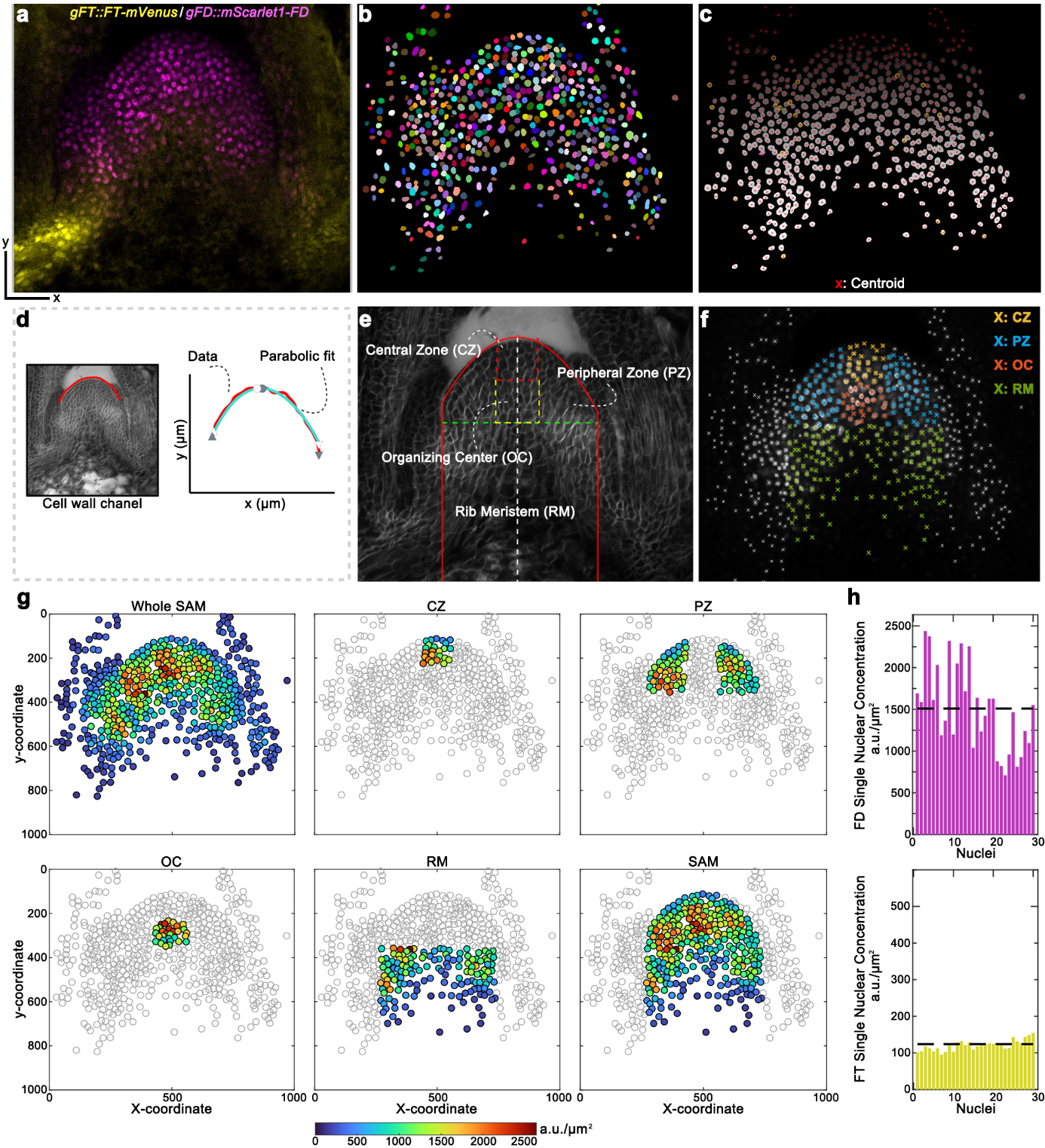


**Supplementary Figure 2**

**Single-cell quantification pipeline for nuclear FD-FT fluorescence signal. a**, Single-image SAM fluorescence confocal microscopy captured from the lateral perspective. The image shows a 11LD-meristem grown under LDs containing *gFT::FT-mVenus* (yellow) *gFD::mScarlet1-FD* (magenta) transgenes. The acquisition parameters are described in Methods. **b**, Nuclear segmentation was performed using *cyto* Cellpose model on the FD channel.tif file. The cell diameter parameter was automatically calibrated. Segmented nuclei are displayed in different colours. **c**, Segmented nuclei in grey with centroid coordinates shown in red. The output nuclear segmentation .png files were processed using custom-made MATLAB code and adapting the previous method^27^ to 2D images. Nuclei were filtered based on size and circularity criteria (valid nuclei satisfy *Area_nucl_*. Є [7,30] in μm_2_ and *Circ*.>=0.7). **d**, A curved line was drawn following the parabolic outline of the SAM in each confocal image (in red). A parabolic fit was then performed, accounting for a possible tilt of the SAM (in green). **e**, A 2D parabolic mask was created based on the fitted parabola. A rectangular mask was also created, extending from the two ends of the parabola up to the inferior edge of the image. These two masks were combined and all intensity values of the pixels outside the new mask were set to 0. **e** and **f**, Using the previously generated parabolic mask and published WUS/CLV3 data [3], the meristematic tissue was divided into four different regions: Central Zone (CZ), Organizing Center (OC), Peripheral Zone (PZ) and Rib Meristem (RB). The centroid of segmented nuclei has a different color for each defined region in (**f**). **g** and **h**, Single-cell nuclear concentrations of FD and FT translational reporters (calculated as the total sum of pixel intensity within a given nucleus divided by its area) were computed for all segmented nuclei in each SAM. **g**, FD spatial single nuclei concentration (a.u./μm2) for different meristematic regions. Coordinates are in pixel. **h**, FD (magenta) and FT (yellow) single nuclear concentration for each nucleus at the OC as an example. The dashed line represents the median value. See Methods for more details.


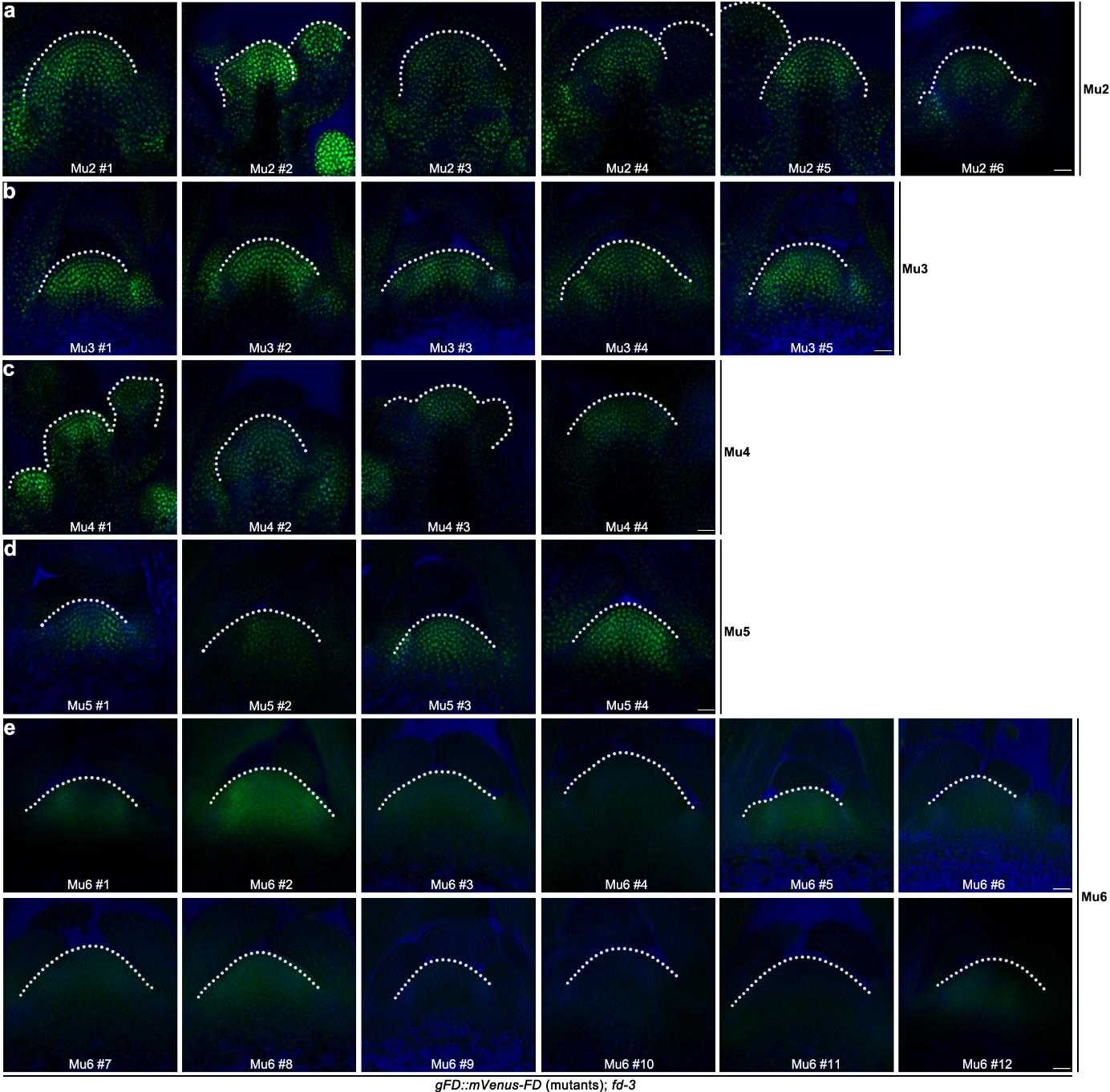


**Supplementary figure 3**

**Confocal imaging of *gFD::mVenus-FD* signals of monomer mutants at the shoot apex. a**−**e**, Confocal imaging of mVenus-FD mutant Mu2, Mu3, Mu4, Mu5 and Mu6, respectively. Scale bars = 20 μm. *n* = 4–12 independent T1 seedlings grown in long days. See also Extended Data Fig. 9.

**
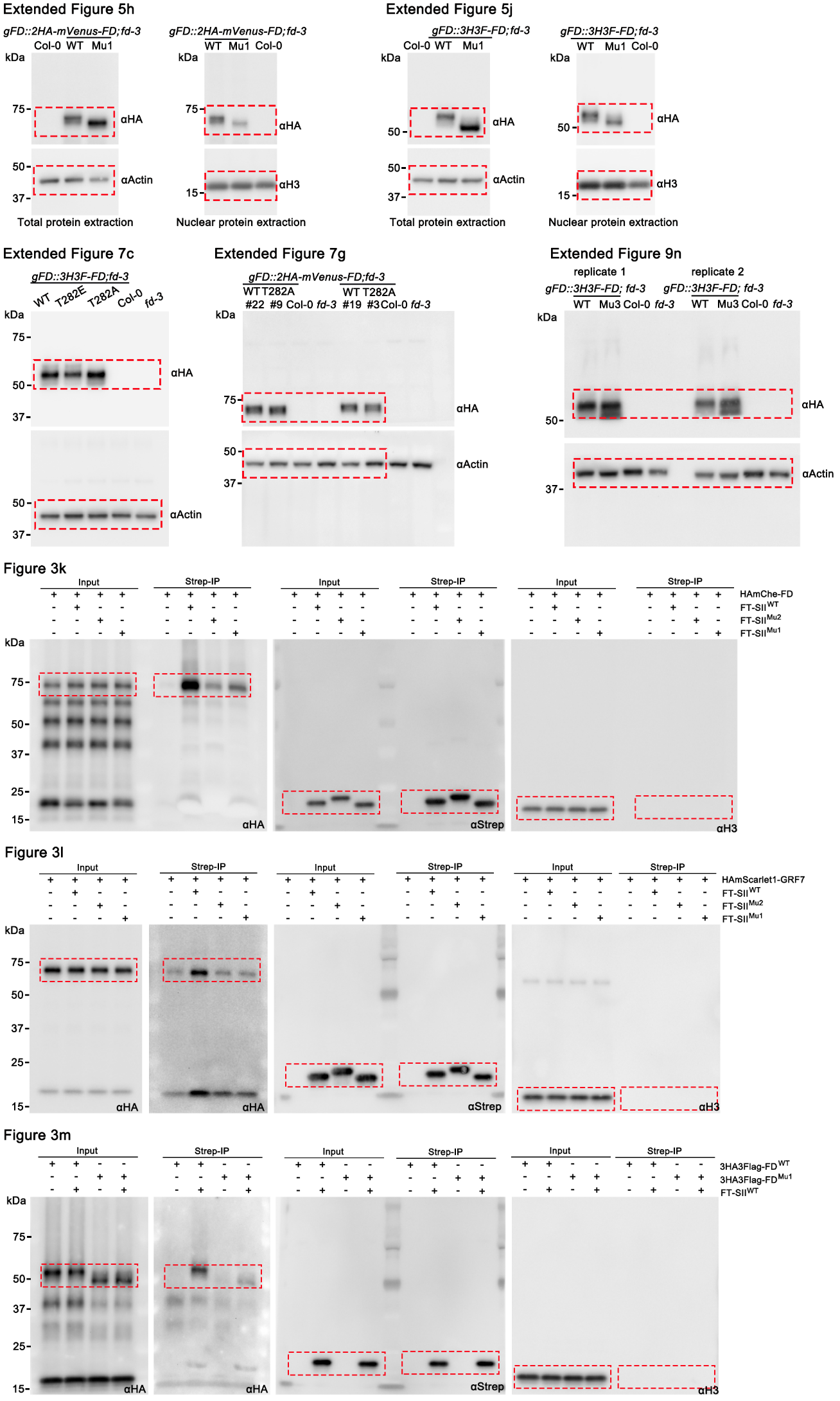
**

**Supplementary figure 4**

**Uncropped western blots**. Samples were run on the same gel as loading controls in Extended Figure 5h,i, Extended Figure 7g and Extended Figure 9n; or on separate gels as sample processing controls in Extended Figure 7c and Figure 3 k,l,m.
