# Supplementary material for: Florigen activation complex forms via multifaceted assembly in Arabidopsis
Source: Nature. 2025 Nov 12;648(8094):686–95. doi: 10.1038/s41586-025-09704-6 (PMC12711580; doi:10.1038/s41586-025-09704-6)
Supplement: Supplementary file 2 — Reporting Summary [file 41586_2025_9704_MOESM2_ESM.pdf]

Corresponding author(s): He Gao and George Coupland
Last updated by author(s): Sep 1, 2025

# Reporting Summary

Nature Portfolio wishes to improve the reproducibility of the work that we publish. This form provides structure for consistency and transparency in reporting. For further information on Nature Portfolio policies, see our Editorial Policies and the Editorial Policy Checklist.

## Statistics

For all statistical analyses, confirm that the following items are present in the figure legend, table legend, main text, or Methods section.

| n/a | Confirmed | |
|---|---|---|
| ☐ | ☒ | The exact sample size ($n$) for each experimental group/condition, given as a discrete number and unit of measurement |
| ☐ | ☒ | A statement on whether measurements were taken from distinct samples or whether the same sample was measured repeatedly |
| ☐ | ☒ | The statistical test(s) used AND whether they are one- or two-sided<br>*Only common tests should be described solely by name; describe more complex techniques in the Methods section.* |
| ☐ | ☒ | A description of all covariates tested |
| ☐ | ☒ | A description of any assumptions or corrections, such as tests of normality and adjustment for multiple comparisons |
| ☐ | ☒ | A full description of the statistical parameters including central tendency (e.g. means) or other basic estimates (e.g. regression coefficient) AND variation (e.g. standard deviation) or associated estimates of uncertainty (e.g. confidence intervals) |
| ☐ | ☒ | For null hypothesis testing, the test statistic (e.g. $F$, $t$, $r$) with confidence intervals, effect sizes, degrees of freedom and $P$ value noted<br>*Give P values as exact values whenever suitable.* |
| ☒ | ☐ | For Bayesian analysis, information on the choice of priors and Markov chain Monte Carlo settings |
| ☐ | ☒ | For hierarchical and complex designs, identification of the appropriate level for tests and full reporting of outcomes |
| ☒ | ☐ | Estimates of effect sizes (e.g. Cohen's $d$, Pearson's $r$), indicating how they were calculated |

*Our web collection on statistics for biologists contains articles on many of the points above.*

## Software and code

Policy information about availability of computer code

| | |
|---|---|
| Data collection | For confocal microscopes, we used Zeiss LSM980 and LSM880.<br>For size exclusion chromatography and multi-angle light scattering (SEC-MALS), we used AKTÄ pure 25M (AKTÄ) coupled to a miniDAWN multi-angle light scattering detector (Wyatt Technology) as well as a refractive index detector (Shodex RI-501). |
| Data analysis | For confocal microscopes, we used Zen 3.10 software (Zeiss).<br>For imaging data processing and analysis, we used Fiji (2.16.0), Cellpose (2.2.3) and Matlab (9.13.0 2022b).<br>For SEC-MALS, we used ASTRA (8.2) software.<br>For analysis of masspectrometry proteomics data we used Perseus version 1.5.8.5.<br><br>Custom code is developed to analyze signal co-localizations at the shoot apex meristem. Source code for analysis signal and figure generation is publicly available at GitHub: https://gitlab.com/GRM_14/gao_ding_et_al_2025/-/tree/011d3d70fc1c0f41670c6f0b860b3c586c8949fd |

For manuscripts utilizing custom algorithms or software that are central to the research but not yet described in published literature, software must be made available to editors and reviewers. We strongly encourage code deposition in a community repository (e.g. GitHub). See the Nature Portfolio guidelines for submitting code & software for further information.

## Data

Policy information about availability of data

All manuscripts must include a data availability statement. This statement should provide the following information, where applicable:

- Accession codes, unique identifiers, or web links for publicly available datasets
- A description of any restrictions on data availability
- For clinical datasets or third party data, please ensure that the statement adheres to our policy

The mass spectrometry proteomics data have been deposited and are accessible at the ProteomeXchange Consortium via the PRIDE78 partner repository with the dataset identifier PXD067955. The full versions of Western blots are shown in Supplementary Fig. 4. The data behind graphs are provided in Supplementary Table 1.

## Research involving human participants, their data, or biological material

Policy information about studies with human participants or human data. See also policy information about sex, gender (identity/presentation), and sexual orientation and race, ethnicity and racism.

| | |
|---|---|
| Reporting on sex and gender | n/a |
| Reporting on race, ethnicity, or other socially relevant groupings | n/a |
| Population characteristics | n/a |
| Recruitment | n/a |
| Ethics oversight | n/a |

Note that full information on the approval of the study protocol must also be provided in the manuscript.

# Field-specific reporting

Please select the one below that is the best fit for your research. If you are not sure, read the appropriate sections before making your selection.

☒ Life sciences ☐ Behavioural & social sciences ☐ Ecological, evolutionary & environmental sciences

For a reference copy of the document with all sections, see nature.com/documents/nr-reporting-summary-flat.pdf

# Life sciences study design

All studies must disclose on these points even when the disclosure is negative.

| | |
|---|---|
| Sample size | Leaf numbers were used to estimate flowering time of Arabidopsis plants (see in figure legends). Sample sizes ($n \geq 10$ per group) were chosen based on established norms in flowering time researches to ensure robust detection of the large effect sizes typical in our studies. Statistical analysis was performed via one-way ANOVA followed by Tukey's pairwise multiple comparison. |
| Data exclusions | No data were excluded. |
| Replication | Flowering time for wild type, mutant and T1 transgenic plants were measured one time for each experiment, and flowering time of crucial transgenic plants were further confirmed one time in T3 homozygous plants with similar results. |
| Randomization | Seedlings were grown randomly in conditional growth chambers and data were collected randomly. |
| Blinding | The investigators were blinded for data collection and/or analysis. |

# Reporting for specific materials, systems and methods

We require information from authors about some types of materials, experimental systems and methods used in many studies. Here, indicate whether each material, system or method listed is relevant to your study. If you are not sure if a list item applies to your research, read the appropriate section before selecting a response.

## Materials & experimental systems

| n/a | Involved in the study |
| --- | --- |
| ☐ | ☒ Antibodies |
| ☒ | ☐ Eukaryotic cell lines |
| ☒ | ☐ Palaeontology and archaeology |
| ☒ | ☐ Animals and other organisms |
| ☒ | ☐ Clinical data |
| ☒ | ☐ Dual use research of concern |
| ☐ | ☒ Plants |

## Methods

| n/a | Involved in the study |
| --- | --- |
| ☒ | ☐ ChIP-seq |
| ☒ | ☐ Flow cytometry |
| ☒ | ☐ MRI-based neuroimaging |

# Antibodies

Antibodies used

The following commercial antibodies were used in this study:
anti-HA (Abcam, ab9110); anti-HA-HRP (Roche, 12013819001, 3F10); anti-ALFA-HRP (NanoTag, N1505, 1G5); anti-Actin (Santa Cruz, sc-47778 HRP, C4); anti-StrepMAB-Classic-HRP (IBA, 2-1509-001); anti-H3-HRP (Abcam, ab1791).

anti-HA (Roche, 12013819001) were used at 1:1000-fold dilution for western blots;
anti-Actin (Santa Cruz, sc-47778) were used at 1:5000-fold dilution for western blots;
anti-Strep (IBA, 2-1509-001) were used at 1:4000-fold dilution for western blots;
anti-ALFA (NanoTag, N1505) were used at 1:2000-fold dilution for western blots;
anti-H3 (Abcam, ab1791) were used at 1:4000-fold dilution for western blots.

Validation

See Methods, all antibodies used in this study are commercially available and have been validated by the manufacturer. Antibody dilutions were used as described above and were optimized with several experiments before the experiment that is reported in this study. Appropriate positive and negative control samples were used in each experiment to ensure antibody specificity.

# Plants

Seed stocks

The Arabidopsis thaliana Columbia (Col-0) ecotype was used as the main experimental organism and were validated from NASC and GABIKat collection. Col-0 (N70000), fd-3 (SALK_054421), fd-10 (GK_290E08).

Novel plant genotypes

Corresponding Col-0, fd-3 or ft-10 mutant plants were grown in the greenhouse under LD conditions and were transformed by the floral dip method using Agrobacterium tumefaciens strain GV3101. The resulting transgenic T1 seeds were screened on half-strength MS medium with supplemented hygromycin for 7 LDs and then they were transferred to soil for the measurement of flowering time.

Authentication

Primers used for plasmid construction for transgenic lines are listed in Supplementary information Table 4.
